# Chemical element profiling in hair of bipolar disorder patients and healthy controls

**DOI:** 10.3389/fphys.2025.1759047

**Published:** 2026-01-28

**Authors:** Hiba Zannadeh, Monica Aas, Vishnu Priya Sampath, Ole Andreassen, Nils Eiel Steen, Kjetil Nordbø Jørgensen, Ofir Tirosh, David Lichtstein

**Affiliations:** 1 Department of Medical Neurobiology, Institute for Medical Research Israel-Canada, Faculty of Medicine, The Hebrew University of Jerusalem, Jerusalem, Israel; 2 Social, Genetic & Developmental Psychiatry Centre, King’s College London, London, United Kingdom; 3 Institute of Clinical Medicine, University of Oslo, Oslo, Norway; 4 Division of Mental Health and Addiction, Oslo University Hospital, Oslo, Norway; 5 Division of Mental Health and Substance Abuse, Diakonhjemmet Hospital, Oslo, Norway; 6 Division of Mental Health and Addiction, Vestre Viken Hospital Trust, Drammen, Norway; 7 Faculty of Science, The Fredy and Nadine Herrmann Institute of Earth Sciences, The Hebrew University of Jerusalem, Jerusalem, Israel

**Keywords:** aluminum, bipolar disorder, boron, brain, cardiac steroids, chemical elements, copper, hair

## Abstract

**Introduction:**

Bipolar disorder (BD) is a severe psychiatric disorder characterized by shifting of mood patterns from manic to depressive episodes. The molecular mechanisms underlying BD have not been fully elucidated, and research into biomarkers is important for prevention and early intervention. The Na^+^, K^+^-ATPase is a metalloprotein that interacts with many chemical elements. It was demonstrated that the interactions of Na^+^, K^+^-ATPase with endogenous cardiac steroids is involved in BD. It was hypothesized that these interactions are mimicked by chemical elements which may participate in BD etiology. We have recently demonstrated that the concentration of Aluminum (Al), Boron (B), Cupper (Cu), Potassium (K), Magnesium (Mg) and Vanadium (V) were significantly lower in the pre-frontal cortex of individuals with BD compared with controls. We hypothesized that differences in the levels of chemical elements between BD and healthy controls would also be reflected in scalp hair.

**Methods:**

To test this hypothesis, the levels of 25 chemical elements were determined by Inductively coupled plasma mass spectrometry (ICP-MS) in the scalp hair of 30 individuals with BD and 30 sex- and age-matched controls.

**Results:**

We found that the levels of Al, Cu, Nickel (Ni) and Thallium (Tl) are elevated in the hair of BD patients compared to controls. In addition, the concentrations of Ni levels in hair samples were correlated with the severity of the mental illness as quantified by the Global Assessment of Functioning Scale.

**Conclusion:**

Although interpretations are tentative due to the limited sample size, our results suggest that changes in chemical elements may be involved either in the etiology of BD or altered due to the disease progression, which needs to be clarified further in larger independent samples.

## 1 Introduction

Bipolar Disorder (BD) is a severe psychiatric illness that affects 1%–4% of the world’s population (Belmaker, 2004; Nierenberg et al., 2023). Individuals with BD suffer from mood oscillations, with episodes of extreme and opposite mood states: mania (or hypomania) and depression, interspaced with periods of steady mood (euthymic phases). A manic episode is characterized by feelings of euphoria, elevated energy levels, decreased need for sleep, racing thoughts, and risk-taking behavior. Depression presents with opposite symptoms: sad mood and low energy levels, disturbed eating and sleeping patterns, feelings of helplessness and low self-esteem, lethargy, anhedonia, suicidal thoughts and behavior (de la Rosa et al., 2017). Psychotic symptoms may also occur as part of mood episodes (Chakrabarti and Singh, 2022). Many molecular pathways and cellular processes have been implicated in BD, including changes in neuroplasticity, mitochondrial function, neurotransmission of GABA and other neurotransmitters, oxidative stress, and inflammation (Kim et al., 2017; Jur et al., 2021). However, the etiology and molecular mechanism of BD are not fully understood, hindering the development of new, targeted therapeutic agents.

Sodium (Na^+^), Potassium (K^+^)-dependent Adenosine Triphosphatase (ATP), (Na^+^, K^+^-ATPase), is the main transporter for Na^+^ and K^+^ across the plasma membrane of all eukaryotic cells (Clausen et al., 2017). The enzyme hydrolyses ATP, and uses the free energy to drive the transport of K^+^ into the cell and Na^+^ out of the cell, against their electrochemical gradients. Hence, the pump function is a key contributor to the asymmetrical distribution of Na^+^ and K^+^ ions and the resting membrane potential. In addition to ion transporting activity the enzyme participates in intracellular signaling (Contreras et al., 2024). Cardiac steroids (CS) such as ouabain, digoxin and bufalin interact with Na^+^, K^+^-ATPase and elicits inhibition or stimulation of its activities (Lichtstein, 1995). In the past 10 years, we and others, utilizing genetic, molecular, behavioral, and pharmacological tools, have substantiated that the Na^+^, K^+^-ATPase and endogenous cardiac steroids are involved in the etiology of BD (Horesh et al., 2024; El-Mallakh et al., 2022). It is suggested that the interaction of endogenous CS with the Na^+^, K^+^-ATPase which causes inhibition of ion transport and activation of intracellular signaling (Horesh et al., 2024) induce metabolic changes that underlie brain dysfunction and mood symptom (El-Mallakh et al., 2022).

The Na^+^, K^+^-ATPase is a metalloprotein requiring Na^+^, K^+^ and Mg^2+^ for ATP hydrolysis (Vasic et al., 2008). Furthermore, many chemical elements have been shown to interact with the Na^+^, K^+^-ATPase and elicit inhibition or stimulation of its activity. These include some first transition series elements like Zinc (Zn^2+^), Nickel (Ni^2+^), Iron (Fe^2+^), Cobalt (Co^2+^) and Cupper (Cu^2+^) and heavy metals like Co^2+^, Mercury (Hg^2+^), Lead (Pb^2+^), Cadmium (Cd^2+^), Manganese (Mn) and Barium (Ba) (Vasic et al., 2008; Yallapragada et al., 2003; Silva et al., 2007; Benders et al., 1994; Kramer et al., 1986). Some elements, like Fe (Costa et al., 2022), Zn and Ni (Haverinen and Vornanen, 2023) exhibits a dual effect on Na^+^, K^+^-ATPase activity, stimulation at low concentrations and inhibition at higher concentrations.

We hypothesized that the binding of chemical elements to the Na^+^, K^+^-ATPase, like the binding of CS, stimulate the cascade leading to behavioral changes and BD. Consequently, we have recently determined the concentration of elements in *postmortem* brain samples from individuals with BD and compared them with matched non-psychiatrically ill controls. The determination of 26 chemical elements in the post-mortem brain tissues revealed that the concentration of Aluminum (Al), Boron (B), Cu, K, Magnesium (Mg) and Vanadium (V) were significantly lower in the prefrontal cortex in BD compared to controls (Sampath et al., 2022a; Sampath et al., 2022b). In addition, the comparison between the elements in the brain in BD and control pointed to B and Al as being involved in the disease (Sampath et al., 2022a).

Previous studies have shown a link between chemical trace elements in human hair and environmental and pathological conditions. For example, significant differences in the scalp-hair trace elements concentration between the coronary artery disease and normal angiogram groups were found for Mg, Calcium (Ca), Chrome (Cr) and Cu (Urbanowicz et al., 2024). Higher iron concentrations in the hair was suggested to be related to higher risk of esophageal squamous cell carcinoma (Hashemian et al., 2023). To the best of our knowledge, only one study measured chemical elements in hair of individuals with BD (Pradeep et al., 2014). That study showed that the concentration of Cu was higher in the hair samples in BD, while the concentrations of Mn, Fe, Zn and Selenium (Se) were lower than the healthy control (HC) group (Pradeep et al., 2014). In the present study, we determined chemical element concentration in scarp hair from individuals with BD and controls, examining the hypothesis that differences previously reported in brain tissue (Sampath et al., 2022a) will also be reflected in scalp hair.

## 2 Materials and Methods

### 2.1 Participants

Patient participants were recruited consecutively from psychiatric units (outpatient and inpatient) from main hospitals in Oslo, as part of the Thematically Organized Psychosis (TOP) Study conducted by the Norwegian Centre for Mental Disorders Research (NORMENT). All participants were recruited from the same catchment area. A total of 30 individuals with BD and 30 healthy controls (HC) were selected for study. Inclusion criteria for the TOP patient groups included having met the criteria for a diagnosis of BD (bipolar I disorder or bipolar II disorder), and being between 18 and 65 years of age. Exclusion criteria included having an organic or substance-induced psychosis, neurological disorder, or unstable or uncontrolled medical conditions thought to interfere with brain function. HC were screened for a history of mental disorder and excluded if they had any lifetime Axis 1 disorders or if they had a first-degree relative with a severe mental disorder. All participants gave written informed consent. The Regional Committee for Medical Research Ethics and the Norwegian Data Inspectorate approved the study.

### 2.2 Clinical assessment

Trained physicians, psychiatrists, and clinical psychologists performed the clinical assessments. Diagnoses were based on the Structured Clinical Interview for DSM-IV Axis I disorders (SCID-I), chapters A-E (Spitzer et al., 1994). All behavioral parameters were assessed quantitatively as previously described (Aas et al., 2023). Age at onset was defined as the first SCID verified episode (D module, affective episode for BD and number of episodes were defined as number of SCID verified affective and psychotic episodes. Clinical symptoms were assessed with the Positive and Negative Syndrome scale (PANSS) (Kay et al., 1987). Function as well as symptom severity was rated using a split version of the Global Assessment of Functioning Scale (GAF; (Pedersen et al., 2007)) dividing into GAF functioning and GAF symptoms. Depressive symptoms were assessed using the Inventory of Depressive Symptoms–Clinician rated scale (IDS-C) (Rush et al., 1986).

### 2.3 Chemical elements extraction

Hair was collected from the posterior vertex region on the head. The hair samples were wrapped in aluminum foil for protection and storage. Sample preparation for elemental analysis was carried out in a clean laboratory in the Institute of Earth Sciences at the Hebrew University. The clean-laboratory includes a monitored positive pressure air supply with HEPA filtration and entirely non-metallic construction. Hair samples were cut with zirconium oxide ceramic scissors into 1.2–1.5 cm segments and washed 3 times with distilled water followed by 3 washes with acetone. The samples were dried by placing the tubes in a hot water bath, under laminar flow of clean air, each sample was weighed. All samples were dissolved in 1:1 distilled HNO_3_: DDW and the acid was evaporated. The remaining powder was dissolved in 5 mL 1% HNO_3_ which was transferred to analysis. Analytical reagent-grade nitric acid (70% wt/wt HNO3) was used for sample and standard preparation. The nitric acid was purified in the laboratory using sub-boiling acid distillation (DST-1000 acid purification system, Savillex).

### 2.4 ICP-MS analysis

Samples were analyzed using an Agilent 8900 ICP-MS QQQ at the Institute of Earth Sciences at the Hebrew University of Jerusalem, calibrated using the MERCK VI multielement standard (Merck). The concentration of 25 elements (Lithium (Li), Na, K, Rubidium (Rb), Mg, Ca, Strontium (Sr), Ba, V, Cr, Mn, Fe, Co, Ni, Cu, Zn, Molybdenum (Mo), Cd, Al, Pb, Arsenic (As), Solver (Ag), Se, Thallium (TL), Uranium (U)) were determined.

An internal standard containing Scandium (Sc), Rhodium (Rh), and Rhenium (Re) was added in-line to all standards and samples for drift and matrix effect corrections. Helium served as the collision gas at a 5 mL/min flow rate. The oxide formation rate (below 1% for Cerium (Ce)) was monitored using a 1 μg/L Ce tuning solution, and sensitivity was optimized prior to analysis. All concentrations were adjusted for the procedural blank values.

### 2.5 Statistical analyses

The demographic and clinical characteristics of the hair samples from patients with BD and HC were analyzed with two-way analysis of variance (ANOVA). Chi-square analysis was used to detect gender differences. As the chemical elements were not normally distributed, a nonparametric test (Mann-Whitney-U) was used to assess differences between groups (case-control status); while demographic continuous variables such as age, and associations with clinical characteristics (age at onset, number of illness episodes, GAF, and IDS were assessed using Spearman’s correlation). Spearman’s correlations were also used to evaluate the inter-element association between element concentrations. The Spearman’s Rank Correlation was used due to the small sample size and because the elements did not have a normal distribution even after log transformation. Analysis of chemical elements in males and females separately was calculated and reported in Supplementary File S1. To reduce type 1 errors, Spearman Correlation Coefficient (rs) equal to or greater than 0.7 in combination with a p value less than 0.005, *cf.* adjusting for the number of elements were selected to be reported. For the case-control comparisons, p-values less than 0.005 were considered significant after adjusting for the number of elements. Analyses were performed in SPSS and in GraphPad Prism v 7.03 (GraphPad Software, Inc.).

## 3 Results

### 3.1 Demographics element concentrations in scalp hair of individuals with BD and matched controls

Demographic characteristics of the control and BD hair samples, gender, age, and ethnicity, are summarized in Table 1. No significant differences in age, gender, or ethnicity were observed between patients and controls. One patient was removed as rediagnosed with schizophrenia, ending up with a total of 29 patients. Twenty-one out of the 29 patients were diagnosed with BD I, and 8 with BD II. The age at onset of depression, psychosis, mania, and hypomania was between 20 and 29 years of age. The mean GAF score of functioning (GAF-F) and symptom levels (GAF-S) were 66 and 64, respectively, indicating current mild symptoms and some difficulty in social, or occupational functioning at the time of assessment. The mean PANSS score was 42, also indicating low symptom load at the time of hair sampling. Nineteen (66%) of the patients were taking medication (antipsychotic medication, mood stabilizers, and/or antidepressants) at the time of the assessment.

**TABLE 1:** Demographics of the hair sample and clinical characteristics of BD patients.

Demographics and clinical characteristics	Patients n = 29	Controls n = 29	Statistics
Age (years), mean ± SD	32.83 ± 10.88	35.79 ± 10.60	t = −1.05, df = 56, p = 0.30
Gender, female yes (%)	20 (69)	16 (55)	X^2^ = 1.17, df = 1, p = 0.28
Ethnicity, European yes (%)	26 (90)	28 (97)	X^2^ = 1.07, df = 1, p = 0.30
Diagnoses BD I, n (%) BD II, n (%)	21 (72.8) 8 (27.6)		
AAO depression (years), mean ± SD	21.71 ± 5.75	**---**	**---**
AAO psychosis (years), mean ± SD	26.40 ± 6.75	**---**	**---**
AAO mania (years), mean ± SD	26.06 ± 6.49	**---**	**---**
AAO hypomania (years), mean ± SD	21.73 ± 5.39	**---**	**---**
At least one suicide attempt, n (%)	7 (29.2)	**---**	**---**
Number of admissions, mean ± SD	2.46 ± 3.04	**---**	**---**
Number of depressive episodes, mean ± SD	7.55 ± 7.44	**---**	**---**
Number of psychotic episodes, mean ± SD	1.68 ± 2.53	**---**	**---**
Number of manic episodes, mean ± SD	2.29 ± 3.52	**---**	**---**
Number of hypomanic episodes, mean ± SD	3.48 ± 6.10	**---**	**---**
GAF-S, mean ± SD	64.92 ± 8.97	**---**	**---**
GAF-F, mean ± SD	66.29 ± 11.82	**---**	**---**
IDS, mean ± SD	12.96 ± 11.59	**---**	**---**
PANSS total score, mean ± SD	42.46 ± 6.81	**---**	**---**
Mediation, n yes (%)	19 (66)	**---**	**---**

BD, bipolar disorders; AAO, age at onset; GAF-S, Global Assessment of Functioning Scale- Symptoms; GAF-F, global assessment of functioning scale; Functioning; IDS, inventory of depressive symptomatology score; PANSS, positive and negative syndrome scale; Medication = antidepressants, mood stabilizers, and/or antipsychotics.

### 3.2 Element concentrations in scalp hair of individuals with BD and controls

**FIGURE 1:**
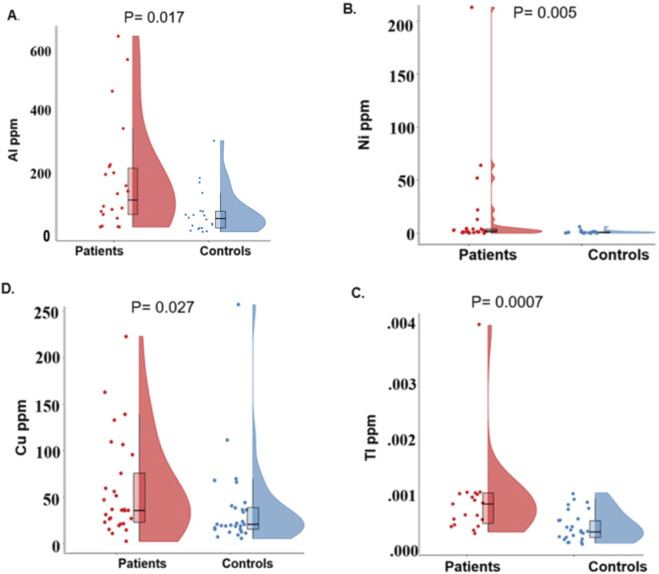
Distribution of Al, Ni, Cu and Tl concentrations in hair samples from BD and non-psychiatrically ill controls **(A–D)**.

A comparison of hair element concentrations between the control and BD groups, using the Mann-Whitney U test, revealed higher concentrations in samples of individuals with BD of four elements before adjusting for the number of elements (Al, Cu, Ni and Tl). The mean value of the four elements were 154% higher than HC for Cu, 250% for Tl, 252% for Al and 1,545% for Ni (Table 2). The distribution of the concentrations of these four elements in BD and in HC hair samples is shown in Figure 1. After adjusting for the number of elements, TI was still statistically higher in patients than in controls. No significant differences were observed for the remaining analyzed elements. No significant correlation between age and AI, Cu, Ni, and TI was observed (p > 0.10).

**TABLE 2:** Element concentrations in hair samples from BD patients and psychiatrically healthy individuals.

Element (ppm)	Control					Bipolar					
	(n)	Mean	SD	Median	Sum of rank	(n)	Mean	SD	Median	Sum of rank	Statistic *p*
Li	29	0.39	0.51	1.2	935	28	0.25	0.34	0.80	718	0.13
Na	26	231	370	759	619	21	161	186	372	510	0.91
Mg	25	74	107	236	593	23	61	60	118	584	0.68
Al	20	67	72	48	336	22	169	178	103	568	0.017*
K	21	80	92	166	373	15	104	135	257	294	0.61
Ca	27	910	1,273	2,709	669	27	1,334	1,676	3,991	816	0.20
V	19	0.021	0.024	0.044	424	22	0.012	0.008	0.019	437	0.51
Cr	11	0.40	0.63	0.91	191	20	0.14	0.16	0.33	306	0.55
Mn	21	0.44	0.88	2.0	504	26	0.24	0.2	0.4	624	1.0
Fe	18	8.4	5.1	10	289	18	11	5.1	14	377	0.16
Co	20	0.015	0.020	0.043	492	26	0.024	0.073	0.19	589	0.63
Ni	18	1.1	1.4	0.8	279	24	16.6	45	2.1	628	0.005*
Cu	29	35	46.0	19	713	29	54	50	33	998	0.027*
Zn	29	250	177	345	872	29	251	203	441	840	0.80
As	22	0.032	0.041	0.1	566	24	0.023	0.021	0.049	515	0.28
Se	29	0.46	0.49	1.4	923	29	2.6	12	32	789	0.30
Rb	29	0.059	0.070	0.15	867	28	0.076	0.12	0.28	786	0.68
Sr	28	1.9	2.2	4	777	28	1.7	1.7	3.0	820	0.72
Mo	24	0.026	0.013	0.03	725	27	0.021	0.018	0.048	602	0.058
Ag	29	0.31	0.37	0.76	906	29	0.49	1	2.0	805	0.43
Cd	29	0.031	0.036	0.1	870	28	0.029	0.034	0.09	784	0.65
Ba	28	0.79	0.84	2.1	756	29	1.0	0.92	1.7	898	0.37
Ti	25	0.0004	0.0003	0.0003	421	0.0008	0.0008	19	0.0008	570	0.0007*
Pb	29	0.89	1.2	1.1	827	29	1.2	1.3	2.5	885	0.65
U	28	0.073	0.12	0.23	890	29	0.023	0.021	0.043	763	0.21

Hair samples were obtained from the psychiatric units from main hospitals in Oslo. Chemical elements were determined as described in Materials and Methods. n-number of samples, out of 30, in which reliable measured concentration of the element was determined. *Nominally significant or significantly higher concentrations compared to control. Cursive = significant after adjusting for number of elements.

The use of mood stabilizers was associated with lower TI levels (yes mood stabilizers, N = 5, mean rank = 4.40 vs. no mood stabilizers, N = 14, mean rank = 12.00, Mann-Whitney U = 7.00, z = −2.63, p = 0.007). The use of antidepressants was associated with lower Cu and TI levels, otherwise, no associations (p > 0.10) were observed between medication and the elements that significantly differed between patients and controls, i.e., AI, Cu, Ni, and TI levels.

Sensitivity analysis investigating elements in males and females separately revealed that the TI group difference was significant in females but not in males (Supplementary File S1).

The distribution of the four elements that were found to be increased in the hair of BD patients compared to controls is shown. The presented plots for Ni and Tl contain all data points, including outliers (values deviating from the average by more than 3 SD). The data for Ni and Tl, without the outliers, are presented in Supplementary File S2.

### 3.3 Inter-element correlations in hair samples from BD and matched control samples

**FIGURE 2:**
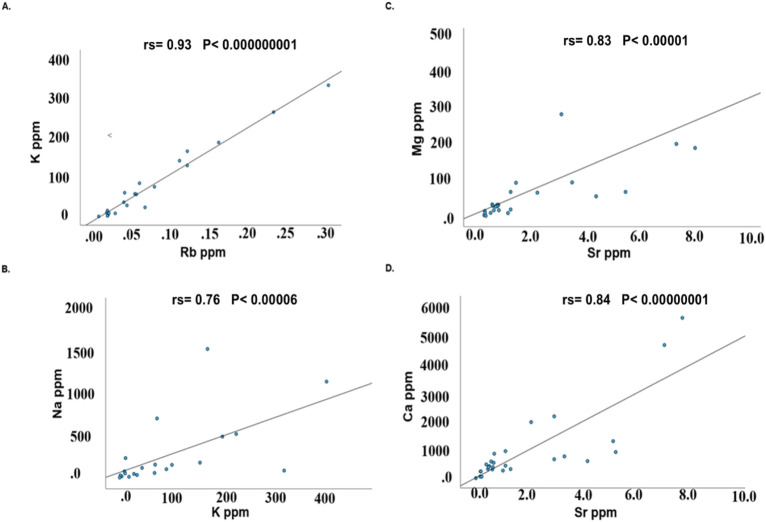
Correlations between elements in control hair samples **(A–D)**.

**FIGURE 3:**
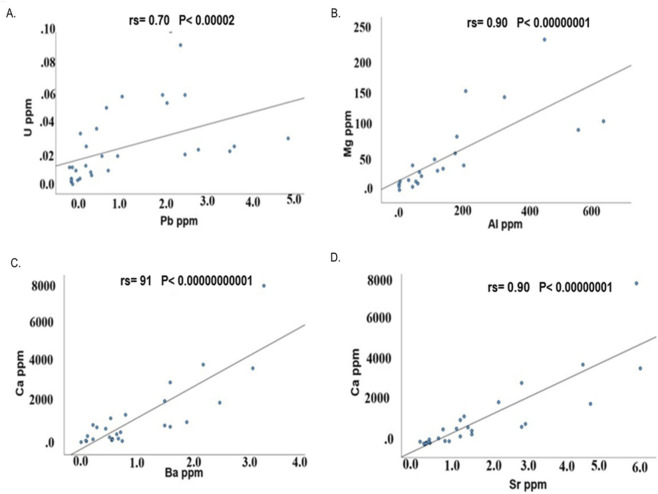
Correlations between elements in hair samples from individuals with BD **(A–D)**.

Spearman’s rank correlations r ≥ 0.7 between element pairs are shown in Table 3. Nine correlations ≥0.7 were detected in control group and 13 in BD samples. Notably, 5 correlations ≥0.7 were detected in both groups (Na-K; K-Rb; Na-Rb; Mg-Sr and Ca-Sr, see Table 3). The four strongest correlations in the control groups (Na-K; K-Rb; Mg-Sr; Ca-Sr) are plotted in Figure 2, and in BD (Ca-Ba; Ca-Sr; Mg-Al; U-Pb) in Figure 3.

**TABLE 3:** Correlation between elements in the hair of BD patients and controls.

	Controls	BD
Element	Correlated with	Correlated with
Na	K, Rb	K, V, Rb
V	Mn	Co
Mg	Sr, Mn, V	Ca, Al, Sr
Ca	Sr	Ba, Sr
K	Rb, Fe	Rb
Mn	-	Ba
Fe	-	Rb
Sr	-	Ba

BD = bipolar disorder; Spearman’s rank correlations were computed between element pairs in hair samples from BD and control subjects. Only the strongest Inter-elemental Spearman’s Rank correlations (rs > 0.7) which were significant after adjusting for multiple testing are shown. The full list of correlations with p < 0.05 is shown in Supplementary File S3.

The correlation between K and Rb (A), Mg and Sr (B), Na and K (C) and Ca and Sr (D) in control hair samples are depicted. These four correlations had the highest Spearman’s rank correlation in this group.

The correlation between U and Pb (A), Mg and Al (B), Ca and Ba (C) and Ca and Sr (D) in control hair samples are depicted. These four correlations had the highest Spearman’s rank correlation in this group.

### 3.4 Elements in hair samples from individuals with BD and clinical characteristics

Several trend-level correlations were observed between elements and clinical characteristics within the BD sample (see Table 4, Supplementary File S4). Patients with a higher number of hypomanic episodes had lower Ba levels. Higher current depressive symptoms from the IDS were associated with lower Zn levels, and more severe symptomatology as assessed with PANSS was associated with lower Ni scores. The strongest trend level correlation was observed between Ni levels and Global Assessment of Functioning Scale, with an association between higher Ni and better functioning (higher score on GAF-F).

**TABLE 4:** Correlation between Element levels in the Hair of individuals with BD and mood parameter.

Mood parameter	Element*
Age at onset of mania Age at onset hypomania Total number of admissions Number of manic episodes Number of hypomanic episodes Global assessment of functioning scale- symptoms Global assessment of functioning scale - function Inventory of depressive symptomatology (IDS) score Positive and negative syndrome scale (PANSS)	V, Fe Ba, Mn, Cd Se Mn, Zn, Ba Mn, -**Ba** Zn **Ni,** Zn -**Zn**, -**Se** Na, -**Ni**, Rb

*Positive correlations are presented in non-boldface and negative correlations in boldface. The presented correlations are without adjusting for multiple testing. After adjusting for multiple testing none of these correlations reached statistical significance levels. The first sentence of the legend indicates the meaning of the bold versus non-bold values.

## 4 Discussion

BD is a chronic disorder characterized by fluctuations in mood state and energy., The molecular mechanism underlining the disease are not fully elucidated and there are currently no valid biomarkers for the disorder (Nierenberg et al., 2023; Kim et al., 2017). Clearly, identifying biomarkers for BD will have significant implications for prevention and early intervention.

In the present study we addressed the possibility that the levels of chemical elements in hair are altered in individuals with BD and that these changes correspond to changes observed in blood and brain samples. The values of the different elements in hair samples in our study were compared to those published recently. A thorough examination of 18 recent available publications has shown that element concentrations in our study, for almost all elements, fall in the range of the published values (Supplementary File S5).

**FIGURE 4:**
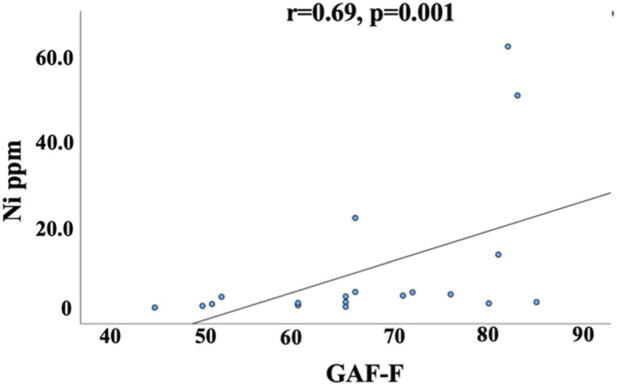
Correlation between Ni levels in hair samples of individuals with BD and GAF-F rating. Spearman’s correlation between Ni levels in hair samples of individuals with BD and GAF F. GAF-F- Global assessment of Functioning Scale Functioning. BD, bipolar disorder. Nominal Trend level correlation with the stricter Bonferroni-adjusted p-value.

Our results demonstrate that at nominal significance threshold, the levels of 4 elements, Al, Cu, Ni and Tl, are increase in the hair in BD compared to the levels in non-psychiatrically ill individuals. After adjusting for the number of elements (Aas et al., 2023) TI was still statistically significant, specifically in females. Inter-element correlations in hair samples from BD and matched control samples revealed 9 strong correlations in the control group and 13 in the BD group (Correlation coefficient r > 0.7). Five correlations were detected in both, the control and BD groups (Na-K; K-Rb; Na-Rb; Mg-Sr and Ca-Sr). In addition, we found several trend level correlations between clinical characteristics and element levels with the strongest trend level correlation between Ni levels and Global Assessment of Functioning Scale which measure the severity of the mental illness (Figure 4).

The composition of hair may provide record on the biochemistry of the organism (Momcilovic et al., 2018). On average, human hairs grow 1 cm per month. Hence, a hair segment 3 cm long from the scalp can be used to determine the chemical events during the preceding 3 months and the hair is used a long-term biological indicator of nutritional status and internal environmental exposure (Wester and van Rossum, 2015; Skalny et al., 2015). Analysis of hair provides useful information regarding drug addiction history or drug toxicity as well as trace elements associated with health and disease (Momcilovic et al., 2018; Philipsen et al., 2021). More recently, measurement of steroids in hair samples has been gaining in popularity (Hakeem et al., 2025; van den Berg et al., 2020). It is tempting to suggest that the identified increased levels of Al, Cu, Ni or Tl may serve as biomarker for BD. Clearly, however, thorough studies on a large number of samples establishing the sensitivity and specificity of an element in BD, will have to be conducted.

Based on our studies on the involvement of Na^+^, K^+^-ATPase and its inhibitors in BD, we hypothesized that chemical elements that interact with this pump may also be involved in the etiology of the disease. The comparison of chemical elements levels in blood and in post-mortem brain samples indeed supported this hypothesis (Sampath et al., 2022a; Sampath et al., 2022b). These studies and the present study revealed not only difference in element levels between BD and controls but also correlations between several elements and BD, suggesting their possible role in the development of BD and their possible use as biomarkers for the disease. These include Al, Cu, B, K, Mg, Ni, Tl and V. As outlined above, the interaction of the elements with Na^+^, K^+^-ATPase may result in the inhibition or stimulation of Na^+^ and K^+^ transport across the plasma membrane and the stimulation of ERK and AKT signaling pathways in neuronal cells (Horesh et al., 2024; El-Mallakh et al., 2022). These, in turn, may alter transcription, oxidative stress, cell growth and neurotransmission, all known to involved in BD (Bezchlibnyk and Young, 2002; Scott and McClung, 2023).

Al and Cu are the two elements that their levels differ in both, brain (lower levels in BD compared to control) and hair (higher levels in BD compared to control), and therefore a more detailed discussion on these two elements is appropriate. Aluminum is the third most abundant element in the Earth’s crust and is widely distributed in the environment but has no known biological function and is not considered essential element (Exley, 2017). Human exposure to aluminum primarily occurs through ingestion, inhalation, and dermal contact (Yokel and McNamara, 2001). In the human body, aluminum is absorbed in small amounts through the gastrointestinal tract, where it binds primarily to plasma proteins such as transferrin and albumin (Krewski et al., 2007). The majority of absorbed aluminum is excreted via the kidneys; thus, individuals with impaired renal function, such as those undergoing dialysis, are at increased risk of aluminum accumulation and toxicity (Alfrey et al., 1976).

Aluminum has been implicated in a variety of neurological disorders that have been associated with an increase in the formation of reactive oxygen species such as Alzheimer’s disease (Exley, 2017; Campbell, 2002). Serum (Zapatero et al., 1995) and hair (Arain et al., 2015a) aluminum levels were shown to be increase in Alzheimer’s disease. Furthermore, studies have shown that hair aluminum levels are higher in samples from patients with schizophrenia compared to controls (Arain et al., 2015b). Our results show that like in AD, Al hair levels are increase compared to control (Table 2). The fact that this increase is found in both neurodegenerative and different psychiatric conditions suggests that increased hair Al is not specific to bipolar disorders.

Cu is an essential microelement found in all living organisms with the unique ability to adopt two different oxidized (Cu^2+^) and reduced (Cu^+^) redox states. It is required for survival and serves as an important catalytic cofactor in redox chemistry for proteins that carry out fundamental biological functions, important in growth and development. An excess of Cu ions in cells is detrimental as these ions can generate free radicals and increase oxidative stress. These are well exemplified by several forms of neurodegenerative diseases, either arising as inherited disorders of Cu metabolism, such as Menkes’ and Wilson’s disease, or as conformational diseases such as Alzheimer’s, Parkinson and prion diseases (Rotilio et al., 2000). In the only study in which chemical elements were measured in hair samples from individuals with BD (Pradeep et al., 2014), the concentration of Cu was found to be higher in hair samples in BD. Our results confirm this observation (Table 2). Furthermore, some studies have shown the levels of Cu were significantly elevated in the serum (Gonzalez-Estecha et al., 2011) and brain *postmortem* samples in BD (Mustak et al., 2010). This suggests that the increased Cu in hair is a reflection of the increased levels in the serum and brain and advocate for closer examination of this element in BD.

In previous study we showed that V levels in the pre-frontal cortex of BD patients are lower than in HC (Sampath et al., 2022a). In addition, the only element that was reduced significantly in the serum of BD patients following treatment, was V (Sampath et al., 2022a). Furthermore, it was demonstrated that V concentrations are higher in the hair of BD patients compared to controls (Naylor et al., 1984). In contrast, in the present study, we did not detect altered V levels in the hair of BD patients (Table 2). Despite the lack of change in hair V in our study, the available literature advocate that the ‘old hypothesis’ of the involvement of V in BD (Sampath et al., 2022b) should be revived and examined.

Another intriguing result is the highly significant increase concentration of Tl in hair of BD patients compared to HC. Although no prior study has directly linked hair Tl levels to BD, Tl is known to accumulate in neural tissues, alter neurotransmitter systems (Tsai et al., 2006), and has been associated with depression, anxiety and cognitive dysfunction in humans (Li et al., 2014). Therefore, our finding of elevated Tl in hair among BD patients is consistent with these observations and raises the possibility that chronic low-level thallium exposure may play a role in mood disorder pathophysiology, which warrants further investigation.

Numerous intercorrelations were detected between element concentrations (Table 3; Figures 2, 3). Ca and Sr were the intercorrelations observed in both BD and in HC. (Figures 2, 3). Interestingly, Ca and Sr intercorrelation was also detected in brain samples from control and individual with BD (Sampath et al., 2022a). These correlations are not surprising since it is established that Sr closely resemble Ca metabolism (Ru et al., 2024).

Systematic examinations of correlations between the concentrations of hair chemical elements and behavior (Table 4) revealed the strongest trend level correlation (r = 0.69) between Ni and clinical characteristics (GAF) in BD (Figure 4), in the direction of higher Ni being associated with better functioning. This is in accord with the observations that Ni administration affects depression- and anxiety-like behavior in animal models (Lamtai et al., 2018). The effects of Ni on dopamine release and glutamate NMDA receptors’ function (Martinez-Martinez et al., 2020) may be mechanisms responsible for Ni effects on behavior.

The small N is a limitation in our study. Future studies should investigate the role of metal elements on clinical characteristics in BD in larger samples. Moreover, the cross-sectional nature of our study design cannot answer causality, only associations between metal elements with illness characteristics and case-control comparisons.

In summary, our findings demonstrate significant alterations in the levels of several chemical elements in the hair of individuals with BD compared to HC. These elemental imbalances may reflect underlying metabolic, environmental, or neurobiological disturbances associated with the disorder. While the cross-sectional nature of this study precludes causal inference, the observed differences highlight the potential relevance of trace element homeostasis in BD pathophysiology. Future studies incorporating larger cohorts, longitudinal designs, and mechanistic investigations are warranted to clarify whether these elemental alterations contribute to disease development, symptom expression, or treatment response.

## Data Availability

The raw data supporting the conclusions of this article will be made available by the authors, without undue reservation.
