# Supplementary material for: Chemical element profiling in hair of bipolar disorder patients and healthy controls
Source: Front Physiol. 2026 Jan 28;16:1759047. doi: 10.3389/fphys.2025.1759047 (PMC12892103; doi:10.3389/fphys.2025.1759047)
Supplement: Supplementary file 1 [file Supplementaryfile1.docx]

**Supplement 1. Element concentrations in the proximal segment of hair samples from individual with BD and psychiatrically healthy individuals in Males and Females.**

**Males**

| **Control BD**  **Element Statistics**  ***(ppm)* (n) Mean S.D median Sum of Rank (n) Mean S.D Median Sum of Rank *p*** | |
| --- | --- |
| **Li**  **Na**  **Mg**  **Al**  **K**  **Ca**  **V**  **Cr**  **Mn**  **Fe**  **Co**  **Ni**  **Cu**  **Zn**  **As**  **Se**  **Rb**  **Sr**  **Mo**  **Ag**  **Cd**  **Ba**  **Tl**  **Pb**  **U** | 13 0.51 0.49 0.27 150 9 0.40 0.70 0.27 104 1.00 12 367 158 284 115 7 246 504 117 75 0.67  11 52 77 22 107 8 57 80 19 84 0.77  8 41 232 78 52 8 194 27 43 84 0.09  10 115 77 145 82 6 117 113 72 54 0.74  11 525 645 434 105 8 679 519 406 86 0.65  9 0.020 0.013 0.012 78 7 0.016 0.026 0.013 58 0.87  7 0.5 0.26 0.078 47 5 0.21 0.76 0.16 31 0.81  10 0.6 0.18 0.16 95 7 0.20 1.2 0.16 58 0.63  8 9.3 8.3 13 50 5 13 4.7 9.0 41 0.38  9 0.016 0.007 0.005 87 8 0.008 0.026 0.008 67 0.60  8 1.6 23 2.8 52 7 11 1.9 1.2 68 0.16  13 26 39 33 135 9 43 19 17 119 0.32  13 277 295 194 152 9 284 197 198 101 0.87  12 0.046 0.031 0.031 129 8 0.038 0.052 0.028 81 0.82  13 0.4 0.68 0.31 157 9 0.52 0.32 0.38 97 0.64  13 0.1 0.08 0.084 135 9 0.11 0.093 0.040 118 0 .33  12 0.9 1.5 0.53 127 8 1.1 0.91 0.54 83 0.94  11 0.029 0.029 0.021 125 8 0.029 0.014 0.025 66 0.23  13 0.2 1.3 0.17 147 9 0.57 0.13 0.15 107 0.84  13 0.033 0.012 0.022 164 8 0.020 0.018 0.030 68 0.14  12 0.5 0.85 0.37 136 9 0.76 0.32 0.45 95 0.78  12 0.0005 0.0003 0.001 92 5 0.000 0.0003 0.001 61 0.09  13 1.1 1.3 1.2 140 9 1.5 1.2 0.83 113 0.53  12 0.0819 0.028 0.022 131 9 0.033 0.14 0.021 100 0.94 |

**Females**

| **Control BD**  **Element statistics**  ***(ppm)*  (n) Mean S.D median Sum of Rank (n) Mean S.D median Sum of Rank  *p*** | |
| --- | --- |
| **Li**  **Na**  **Mg**  **Al**  **K**  **Ca**  **V**  **Cr**  **Mn**  **Fe**  **Co**  **Ni**  **Cu**  **Zn**  **As**  **Se**  **Rb**  **Sr**  **Mo**  **Ag**  **Cd**  **Ba**  **Tl**  **Pb**  **U** | 16 0.30 0.23 0.11 339 19 0.18 0.27 0.29 292 0.094  14 115 189 59 210 14 118 131 70 197 0.77  14 92 52 42 209 15 64 124 43 227 0.95  12 84 146 127 132 14 155 88 48 219 0.12  11 48 167 17 110 9 95 55 23 101 0.65  16 1174 1904 983 260 19 1610 1565 570 371 0.35  10 0.023 0.006 0.008 138 15 0.011 0.025 0.01 187 0.65  4 0.13 0.11 0.071 44 15 0.11 0.095 0.11 146 0.69  11 0.30 0.21 0.18 161 19 0.25 0.44 0.13 304 0.68  10 7.7 3.4 8.6 97 13 9.7 5.6 6.5 180 0.14  11 0.015 0.087 0.007 172 18 0.032 0.015 0.01 264 0.77  10 0.73 51 1.4 85 17 19 0.78 0.52 293 0.006*  16 42 54 33 241 20 59 59 19 426 0.077  16 228 152 181 298 20 236 163 180 368 0.95  10 0.016 0.011 0.014 143 16 0.016 0.007 0.015 209 0.69  16 0.50 14 0.31 322 20 3.6 0.6 0.32 344 0.41  16 0.041 0.13 0.016 321 19 0.061 0.038 0.022 309 0.27  16 2.7 1.7 1.3 304 20 1.9 2.6 1.1 362 0.80  13 0.023 0.009 0.015 239 19 0.017 0.012 0.022 290 0.36  16 0.43 0.83 0.096 337 20 0.45 0.46 0.22 329 0.19  16 0.03 0.040 0.025 276 20 0.033 0.047 0.016 390 0.52  16 0.99 0.95 0.78 271 20 1.1 1 0.47 395 0.43  13 0.0003 0.0009 0.0007 110 14 0.000 0.0002 0.003 268 0.000*  16 0.73 1.3 0.51 272 20 1.1 1.1 0.34 394 0.44  16 0.067 0.016 0.011 336 20 0.019 0.1 0.022 330 0.20 |
