# Supplementary material for: Chemical element profiling in hair of bipolar disorder patients and healthy controls
Source: Front Physiol. 2026 Jan 28;16:1759047. doi: 10.3389/fphys.2025.1759047 (PMC12892103; doi:10.3389/fphys.2025.1759047)
Supplement: Supplementary file 2 [file Supplementaryfile5.docx]

| **Source** | **Li** | **Na** | **Mg** | **Al** | **K** | **Ca** | **V** | **Cr** | **Mn** | **Fe** | **Co** | **Ni** | **Cu** | **Zn** | **As** | **Se** | **Rb** | **Sr** | **Mo** | **Ag** | **Cd** | **Ba** | **Tl** | **Pb** | **U** |
| --- | --- | --- | --- | --- | --- | --- | --- | --- | --- | --- | --- | --- | --- | --- | --- | --- | --- | --- | --- | --- | --- | --- | --- | --- | --- |
| **Our results** | 0.39 | 231 | 74 | 66 | 80 | 909 | 0.021 | 0.39 | 0.44 | 8.4 | 0.015 | 1.1 | 35 | 250 | 0.032 | 0.46 | 0.059 | 1.9 | 0.025 | 0.31 | 0.031 | 0.79 | 0.0004 | 0.89 | 0.073 |
| **(1)** |  |  | 39 |  |  | 494 | 0.001 | 0.11 | 0.016 | 11 | 0.004 |  | 9 | 129 |  | 0.37 |  |  |  |  |  |  |  |  |  |
| **(2)** |  | 142 | 28 | 7.7 | 34.5 | 406 | 0.13 | 0.21 | 0.159 | 9.5 | 0.016 | 0.28 | 106 | 483 | 0.034 | 0.55 |  | 4.7 | 0.051 | 0.001 | 0.045 | 0.35 | 0.0003 | 2.2 |  |
| **(3)** |  |  |  |  |  |  |  | 0.88 | 3.5 |  |  | 5.9 | 21 | 149 |  |  |  |  |  |  | 0.59 |  |  | 3.3 |  |
| **(4)** |  | 68 | 50 | 13 | 59 | 96 | 0.027 | 1.8 | 0.39 | 16 | 0.015 | 0.43 | 15 | 269 | 0.075 | 0.27 | 0.056 | 2.3 | 0.034 | 0.035 | 0.014 | 1.2 | 0.004 | 0.47 | 0.098 |
| **(5)** |  | 88 | 26 | 8 | 279 |  |  |  |  | 7.1 |  |  | 9.9 | 51 |  |  |  |  |  |  | 0.0001 |  |  | 0.96 |  |
| **(6)** |  |  |  |  |  |  |  |  | 0.53 | 20 | 0.014 | 10 |  | 185 |  | 0.47 |  |  |  |  |  |  |  |  |  |
| **(7)** |  | 120 | 57 |  | 60 | 810 |  | 0.4 | 0.18 | 7 | 19 |  |  | 160 |  | 0.5 |  |  |  |  |  |  |  |  |  |
| **(8)** | 405 | 43 | 18 |  | 467 | 320 |  | 0.34 | 0.45 | 13 | 0.02 | 0.45 | 32 | 135 |  | 0.58 |  |  | 0.044 |  | 0.058 | 0.58 | 0.005 | 1.7 |  |
| **(9)** |  |  |  | 445 |  | 3301 |  | 100 |  | 707 | 3.8 |  | 12 | 482 |  | 0.32 |  |  |  |  | 0.15 |  |  | 4.4 |  |
| **(10)** |  |  |  |  |  |  |  |  |  | 23 |  |  | 10 | 245 | 0.55 | 0.66 |  |  |  |  | 0.014 |  |  | 0.34 |  |
| **(11)** |  |  |  |  |  |  |  | 0.55 |  |  |  |  | 9 |  | 0.24 |  |  |  |  |  | 0.11 |  | 12 |  |  |
| **(12)** |  |  |  |  |  |  |  |  | 1.3 | 20 |  | 0.28 | 13 | 257 |  | 4.8 |  | 48 |  |  |  |  |  |  |  |
| **(13)** |  |  | 20 | 6.9 |  | 297 | 0.017 | 0.12 | 0.21 | 12 | 0.007 | 0.15 | 10 | 153 | 0.031 | 0.44 |  |  |  |  | 0.021 |  |  |  |  |
| **(14)** |  |  |  | 3 |  |  | 0.04 | 0.35 |  |  | 0.004 | 0.055 | 13 | 195 |  |  |  |  |  | 0.03 |  |  |  |  |  |
| **(15)** | 6 | 7605 | 787 |  | 154 |  |  |  | 21 | 275 |  |  | 13 | 257 |  | 4.8 |  | 48 |  |  |  |  |  |  |  |
| **(16)** | 0.09 |  |  | 32 |  |  | 1.1 | 0.67 | 1.9 | 12 | 4.4 | 5.1 | 77 | 413 | 3.4 | 1.2 | 0.05 |  | 1 | 1.27 | 3.5 | 3.6 |  | 13 | 0.24 |
| **(17)** |  |  | 305 |  |  | 1952 |  |  |  |  |  |  |  |  |  | 1.8 |  | 6.4 |  |  |  | 4.4 |  |  |  |
| **(18)** |  |  |  | 48 |  |  | 0.22 | 1.1 | 2.8 | 66 | 0.078 | 2.5 | 16 | 269 | 0.07 | 0.39 |  | 0.14 |  | 0.33 |  |  | 6.5 | 0.12 |  |

**Supplement 5.**

**The Levels of Chemical Elements in Hair -Literature Survey**

Literature survey on studies in which hair chemical elements were determined. The mean of the elements concentration (ppm) in recent 18 studies are included in the survey. The levels in the hair obtained in the present study are depicted on the first line of the Table (Our results).

1. Kirichuk AA, Skalny AV, Schaumloffel D et al. Assessment of trace element and mineral levels in students from Turkmenistan in comparison to Iran and Russia. J Trace Elem Med Biol 2024;84:127439.

2. Ruiz R, Estevan C, Estevez J, Alcaide C, Sogorb MA, Vilanova E. Reference Values on Children's Hair for 28 Elements (Heavy Metals and Essential Elements) Based on a Pilot Study in a Representative Non-Contaminated Local Area. Int J Mol Sci 2023;24.

3. Dai L, Deng L, Wang W et al. Potentially toxic elements in human scalp hair around China's largest polymetallic rare earth ore mining and smelting area. Environ Int 2023;172:107775.

4. Xia YY, de Seymour JV, Yang XJ et al. Hair and cord blood element levels and their relationship with air pollution, dietary intake, gestational diabetes mellitus, and infant neurodevelopment. Clin Nutr 2023;42:1875-1888.

5. Kusanagi E, Takamura H, Hoshi N, Chen SJ, Adachi M. Levels of Toxic and Essential Elements and Associated Factors in the Hair of Japanese Young Children. Int J Environ Res Public Health 2023;20.

6. Umarova G, Batyrova G, Tlegenova Z et al. Essential Trace Elements in Scalp Hair of Residents across the Caspian Oil and Gas Region of Kazakhstan. Toxics 2022;10.

7. Lee YA, Kim HN, Song SW. Associations between Hair Mineral Concentrations and Skeletal Muscle Mass in Korean Adults. J Nutr Health Aging 2022;26:515-520.

8. Ambeskovic M, Laplante DP, Kenney T et al. Elemental analysis of hair provides biomarkers of maternal hardship linked to adverse behavioural outcomes in 4-year-old children: The QF2011 Queensland Flood Study. J Trace Elem Med Biol 2022;73:127036.

9. Ooi TC, Singh DKA, Shahar S et al. Higher Lead and Lower Calcium Levels Are Associated with Increased Risk of Mortality in Malaysian Older Population: Findings from the LRGS-TUA Longitudinal Study. Int J Environ Res Public Health 2022;19.

10. Ren M, Zhao J, Wang B et al. Associations between hair levels of trace elements and the risk of preterm birth among pregnant Wwomen: A prospective nested case-control study in Beijing Birth Cohort (BBC), China. Environ Int 2022;158:106965.

11. Yang M, Xu Y, Ke H, Chen H. Cumulative Effect and Content Variation of Toxic Trace Elements in Human Hair around Xiaoqinling Gold Mining Area, Northwestern China. Int J Environ Res Public Health 2021;18.

12. Qin Y, Xu C, Li W et al. Metal/metalloid levels in hair of Shenzhen residents and the associated influencing factors. Ecotoxicol Environ Saf 2021;220:112375.

13. Tinkov AA, Skalnaya MG, Simashkova NV et al. Association between catatonia and levels of hair and serum trace elements and minerals in autism spectrum disorder. Biomed Pharmacother 2019;109:174-180.

14. Merino JJ, Parmigiani-Izquierdo JM, Toledano Gasca A, Cabana-Munoz ME. The Long-Term Algae Extract (Chlorella and Fucus sp) and Aminosulphurate Supplementation Modulate SOD-1 Activity and Decrease Heavy Metals (Hg(++), Sn) Levels in Patients with Long-Term Dental Titanium Implants and Amalgam Fillings Restorations. Antioxidants (Basel) 2019;8.

15. Janbabai G, Alipour A, Ehteshami S, Borhani SS, Farazmandfar T. Investigation of Trace Elements in the Hair and Nail of Patients with Stomach Cancer. Indian J Clin Biochem 2018;33:450-455.

16. Tamburo E, Varrica D, Dongarra G, Grimaldi LM. Trace elements in scalp hair samples from patients with relapsing-remitting multiple sclerosis. PLoS One 2015;10:e0122142.

17. Kato M, Kumasaka MY, Ohnuma S et al. Comparison of Barium and Arsenic Concentrations in Well Drinking Water and in Human Body Samples and a Novel Remediation System for These Elements in Well Drinking Water. PLoS One 2013;8:e66681.

18. Al-Sabbak M, Sadik Ali S, Savabi O, Savabi G, Dastgiri S, Savabieasfahani M. Metal contamination and the epidemic of congenital birth defects in Iraqi cities. Bull Environ Contam Toxicol 2012;89:937-44.

**References**
