# Supplementary material for: Chemical element profiling in hair of bipolar disorder patients and healthy controls
Source: Front Physiol. 2026 Jan 28;16:1759047. doi: 10.3389/fphys.2025.1759047 (PMC12892103; doi:10.3389/fphys.2025.1759047)
Supplement: Supplementary file 3 [file Supplementaryfile3.docx]

**Supplement 3 - Correlation Between Elements in Hair of individuals with BD and Matched Controls.**

| **Element** | **Control** | **Bipolar Disorder** |
| --- | --- | --- |
| **Li**  **Na**  **Mg**  **Al**  **K**  **Ca**  **V**  **Cr**  **Mn**  **Fe**  **Co**  **Ni**  **Cu**  **Zn**  **As**  **Se**  **Rb**  **Sr**  **Mo**  **Ag**  **Cd**  **Ba**  **Tl**  **Pb**  **U** | As, Rb  *K*, V, Zn, Rb, Mo, Cd, Pb  Ca, V, Mn, Fe, *Sr*  -  *Na*, Fe, *Rb*, Pb  Mg, **-As**, *Sr*, Ba  Na, Mg, Mn, Fe, Se, Mo, Ag  Co  Mg, V, Fe, Co, Zn, Sr, Pb, U  Mg, K, V, Mn, Pb  Cr, Mn, Se, Ag, Ba  Pb  **-As**, Sr, Ag, Ba, Pb  Na, Mn  Li, **-Ca, -Cu, -Sr, -Ag, -Ba**  V, Co, Mo  Li, *Na*, *K*, Mo, Cd, Ti  *Mg,* *Ca*, Mn, Cu, **-As**, Ba  Na, V, Se, Rb, U  V, Co, Cu, **-As**, Ba  Na, Rb  Ca, Co, Cu, **-As**, Sr, Ag, Pb  Rb  Na, K, Mn, Fe, Ni, Cu, Ba, U  Mn, Mo, Pb | Se, Rb  *K,* V, Cr, Fe, As, Rb, Mo  *Al,* Co, *Ca*, *Sr*  *Mg*, Ca, Sr  *Na*, V, *Rb*  *Mg*, Al, Mn, Co, Cu, *Sr*, *Ba*  Na, K, Cr, Fe, *Co*, Mo, Ag, Pb  Na, V, Pb  Ca, **-Ni**, Cu, Sr, Ag, Cd, Ba  Na, V, Rb, Ag  Mg, Ca, *V,* Sr, Cd, Ba  **-Mn**, Zn, Se  Ca, Mn, Sr, Ag, Cd, Ba, Pb, U  Ni, As, Se, Mo, Pb, U  Na, Zn, Se, Rb, Pb  Li, Ni, Zn, As, *Mo*, Pb  Li, *Na*, *K*, Fe, As, Mo  Mg, Al, Ca, Mn, Co, Cu, Cd, Ba  Na, V, Zn, *Se*, Rb, *Pb*  V, Mn, Fe, Cu, Cd, Ba, Pb  Mn, Co, Cu, Sr, Ag, *Ba,* Pb  *Ca*, *Mn*, Co, Cu, Sr, Ag, Cd  -  V, Cr, *Cu*, Zn, As, Se, *Mo*, Ag, Cd, U  Cu, Zn, *Pb* |

Bold= Negative correlations.

Cursive with underscore= Correlations significant after adjusting for multiple testing (25x25 analysis).
