# Supplementary material for: Chemical element profiling in hair of bipolar disorder patients and healthy controls
Source: Front Physiol. 2026 Jan 28;16:1759047. doi: 10.3389/fphys.2025.1759047 (PMC12892103; doi:10.3389/fphys.2025.1759047)
Supplement: Supplementary file 4 [file Supplementaryfile2.docx]

**Supplement 2. The distribution of the four elements that were found to be increased in the hair of BD patients compared to controls is shown. The presented plots for Ni and Tl contain the experimental data without outliers (values deviating from the average by more than 3 SD).**


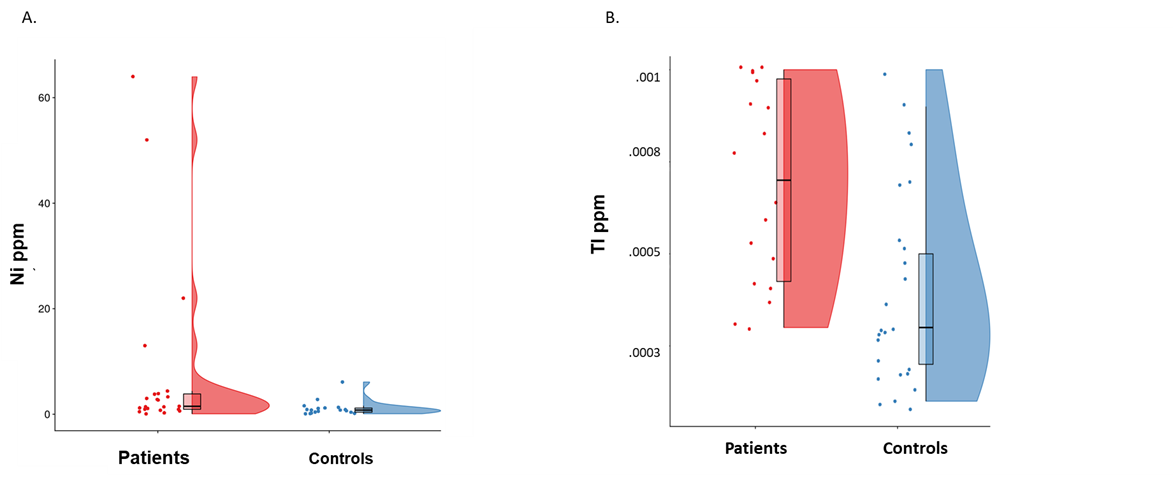
Mann Whitney U test=121, z=-2.41, p=0.016 Mann Whitney U test=109, z=-3.07, p=0.002
