# Supplementary material for: Chemical element profiling in hair of bipolar disorder patients and healthy controls
Source: Front Physiol. 2026 Jan 28;16:1759047. doi: 10.3389/fphys.2025.1759047 (PMC12892103; doi:10.3389/fphys.2025.1759047)
Supplement: Supplementary file 5 [file Supplementaryfile4.docx]

**Supplement 4. Correlation between element levels and mood parameter in Male and Female BD patients**

|  | Male | Female |
| --- | --- | --- |
| Age at onset of Mania  Age at onset of Hypomania  Number of manic episodes  Number of hypomanic episodes  Total number of admissions  Global Assessment of Functioning Scale- Function.  Global Assessment of Functioning Scale- Symptoms.  Inventory of Depressive Symptomatology (IDS) score  Positive and Negative Syndrome Scale (PANSS) | K, Ag, Fe, Ag, Cd, Ba  Cr  K, Ca, V, Mn, Rb, Ba  Ni  -**Ni**, -**Se**, -**Mo** | Fe, Co  Ca, -**Se**, Sr,-**Mo**, Ba  Li, -**Ni**  Cu, Pb  Fe, Ni, Rb  **-Fe**, -**Rb**  Na, Fe, -**Ni**, Rb |

No analyses survived adjusting for multiple testing. Elements that are significantly correlated with mood, before adjusting for multiple testing are depicted in bold.
